# Acupuncture for pediatric patients with cardiogenic stroke sequelae: A CARE-compliant case report

**DOI:** 10.1097/MD.0000000000044948

**Published:** 2025-10-03

**Authors:** Ruiqiao Guan, Luling Wang, Jing Chen, Shiyu Su, Wei Liu

**Affiliations:** a Department of Chinese Medicine, The First Affiliated Hospital of Dalian Medical University, Dalian City, Liaoning Province, China; b Faculty of Chinese Medicine, Macau University of Science and Technology, Macau, China; c Department of Rehabilitation Medicine, The First Affiliated Hospital of Dalian Medical University, Dalian City, Liaoning Province, China.

**Keywords:** acupuncture, cardiogenic stroke, case report, Panlong needling technique, pediatric strokes

## Abstract

**Rationale:**

Pediatric strokes frequently result from underlying heart conditions. Early diagnosis and treatment are essential for restoring neurological function in patients and enhancing their quality of life. Currently, there are no reports on acupuncture treatment for pediatric strokes. We report a case of a patient in the poststroke phase of cardiogenic stroke. This child was found to have a deficiency in the oval fossa of the interatrial septum and exhibited clinical symptoms, such as head bobbing and impaired movement of the right limbs, following surgical events.

**Patient concerns:**

A 4-year-old girl with a cardiogenic stroke (late stage) presented to our clinic with weakness in the right upper limb and right-hand apraxia.

**Diagnoses:**

The diagnosis of pediatric cardiogenic stroke (poststroke phase) was confirmed through physical examination, imaging, and medical history. In this case, the Traditional Chinese Medicine diagnosis is classified as stroke (obstruction of phlegm and blood stasis).

**Interventions:**

Based on the patient’s condition, we used the Jiaji acupoint with the Panlong needling technique combined with a modified “Xingnao Kaiqiao” acupuncture therapy.

**Outcomes:**

After 5 months of treatment, the child’s impaired movement of the right limbs and hand function improved significantly.

**Lessons:**

This case suggests that acupuncture can be an effective method for treating poststroke sequelae in children.

## 1. Introduction

The incidence of stroke in children is between 3 and 25 per 100,000, with ischemic and hemorrhagic strokes occurring equally.^[1]^ This makes stroke one of the leading causes of disability and death in this population. Heart disease is a major cause of stroke in children, with cardiac embolic strokes accounting for about 30% of all pediatric strokes, which can be caused by congenital heart disease, surgical-related events, or acquired heart disease.^[2]^ Research indicates that children with heart disease who experience acute ischemic stroke often have their strokes begin in infancy. They are more prone to develop “cardiac embolic strokes,” which typically present as multiple bilateral strokes that may affect both anterior and posterior circulation simultaneously.^[3]^ Quick diagnosis of strokes in children is essential for improving neurological recovery, managing complications, and preventing secondary strokes. Unfortunately, there are very few clinical treatment options available for addressing poststroke complications in children. Acupuncture, a Traditional Chinese Medicine (TCM) treatment, plays an important role in alleviating neurological deficits after stroke,^[4,5]^ but there are few reports on acupuncture treatment for pediatric stroke cases.

## 2. Case presentation

**Figure 1.**
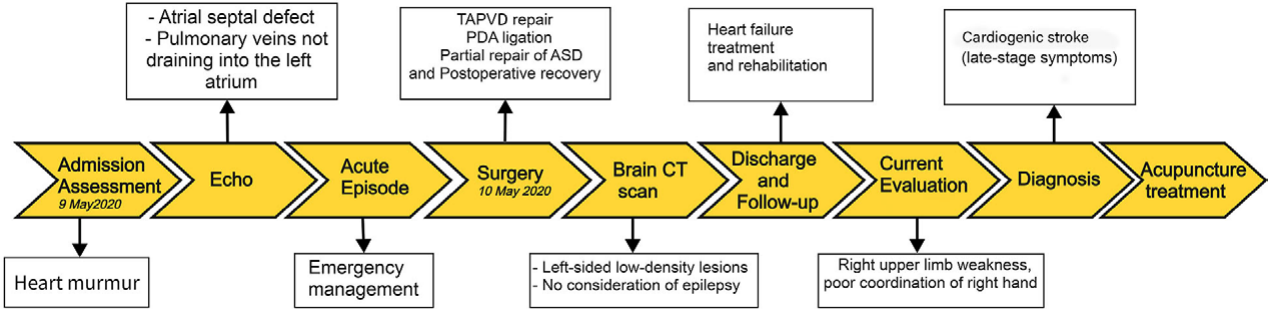
Diagnosis and treatment process. ASD = atrial septal defect, PDA = patent ductus arteriosus, TAPVD = total anomalous pulmonary venous drainage.

We present a case of a 4-year-old girl with right upper limb weakness and an inability to grip with her right hand for over 3 years. The patient was admitted for observation and treatment on May 9, 2020, due to a heart murmur was detected during a physical examination. On the same day, an echocardiogram revealed enlargement of the right atrium and right ventricle, and small left atrium and left ventricle; a 7mm defect in the oval fossa of the atrial septum, accompanied by a right-to-left shunt. The pulmonary veins failed to drain into the left atrium; instead, a common trunk was observed draining into the left branch of the portal vein, resulting in accelerated blood flow in the inferior vena cava. On the night of May 9, 2020, the patient suddenly cried out, and her complexion and limbs were observed to be cyanotic. Cardiac monitoring showed a heart rate fluctuating between 140 and 160 beats per minute, and oxygen saturation fluctuating around 20% to 30%. At the same time, the patient exhibited poor responsiveness, a cyanotic complexion, rapid breathing at 60 breaths per minute, perioral cyanosis, and coarse breath sounds in both lungs. A II/VI systolic murmur was detected at the left second intercostal space, the second heart sound was fixedly split, and both lower limbs displayed a marbled appearance with cool extremities. After resuscitation, the oxygen saturation improved to between 80% and 84%. However, the patient’s cardiac function further deteriorated, and on May 10, 2020, she underwent total anomalous pulmonary venous drainage surgery, ligation of the patent ductus arteriosus, and partial repair of the atrial septal defect (SDA) under general anesthesia and hypothermic cardiopulmonary bypass (CPB). On May 12, 2020, delayed chest closure was performed, and postoperatively, she continued to receive ventilatory support, peritoneal dialysis, positive inotropic and diuretic therapy, myocardial nutrition, and symptomatic antiinfective treatment. The patient’s condition gradually improved, then was transferred to a general ward. On the same day she exhibited lateral head shaking, and a cranial CT suggested brain atrophy, low-density lesions in the left parietal lobe, and high-density lesions in the surrounding cerebral cortex. During that time, epilepsy was not considered, and no special treatment was given. She has been followed up in the cardiology and rehabilitation departments for nearly 3 years (The diagnosis and treatment process is shown in Fig. 1).

**Figure 2.**
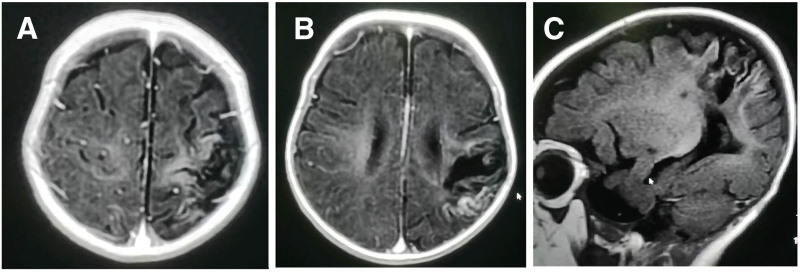
Cranial MRI: There is a loss of the left frontal parietal lobe, insula, and the left central light gray matter, with irregular morphology of the local gyri. Irregular long T1 and long T2 signals are observed, with relatively clear boundaries, measuring approximately 31mm42mm26mm.

During the outpatient examination in June 2024, the followings were observed: The patient was conscious, with stable breathing, and responsive to sound, light, and the environment. The head followed along the extension line of the trunk when lifted from the supine position; and the head could be lifted in the prone position, with elbows supporting the body, but could not be supported with the hands. The parachute sign was negative. There was a slight forward lean in the sitting position. The child’s upper limbs had difficulty bearing weight while standing, and the movement of the right upper limb was less than that of the left. The right upper limb muscle strength was III+, and was normal in the other limbs; however, the right thumb was in a tense adducted position, and the right hand had almost no active grasping. The Gross Motor Function Measure (GMFM) score showed that the child had hand dysfunction, characterized by weak grasping ability, inadequate force for tasks, and poor hand-eye coordination, which indicated hand apraxia. The follow-up magnetic resonance imaging (MRI) of the head revealed localized cerebral softening and atrophy in the left temporoparietal lobe, insula, and precentral gyrus. The range of the softening lesion was larger than before, and a small amount of fluid under the left parietal subdural membrane had been absorbed compared to the previous examination. The bilateral frontal and temporal subarachnoid spaces were prominent (Fig. 2). The diagnosis was a cardiogenic stroke (late stage). The attending physician considered acupuncture treatment.

**Figure 3.**
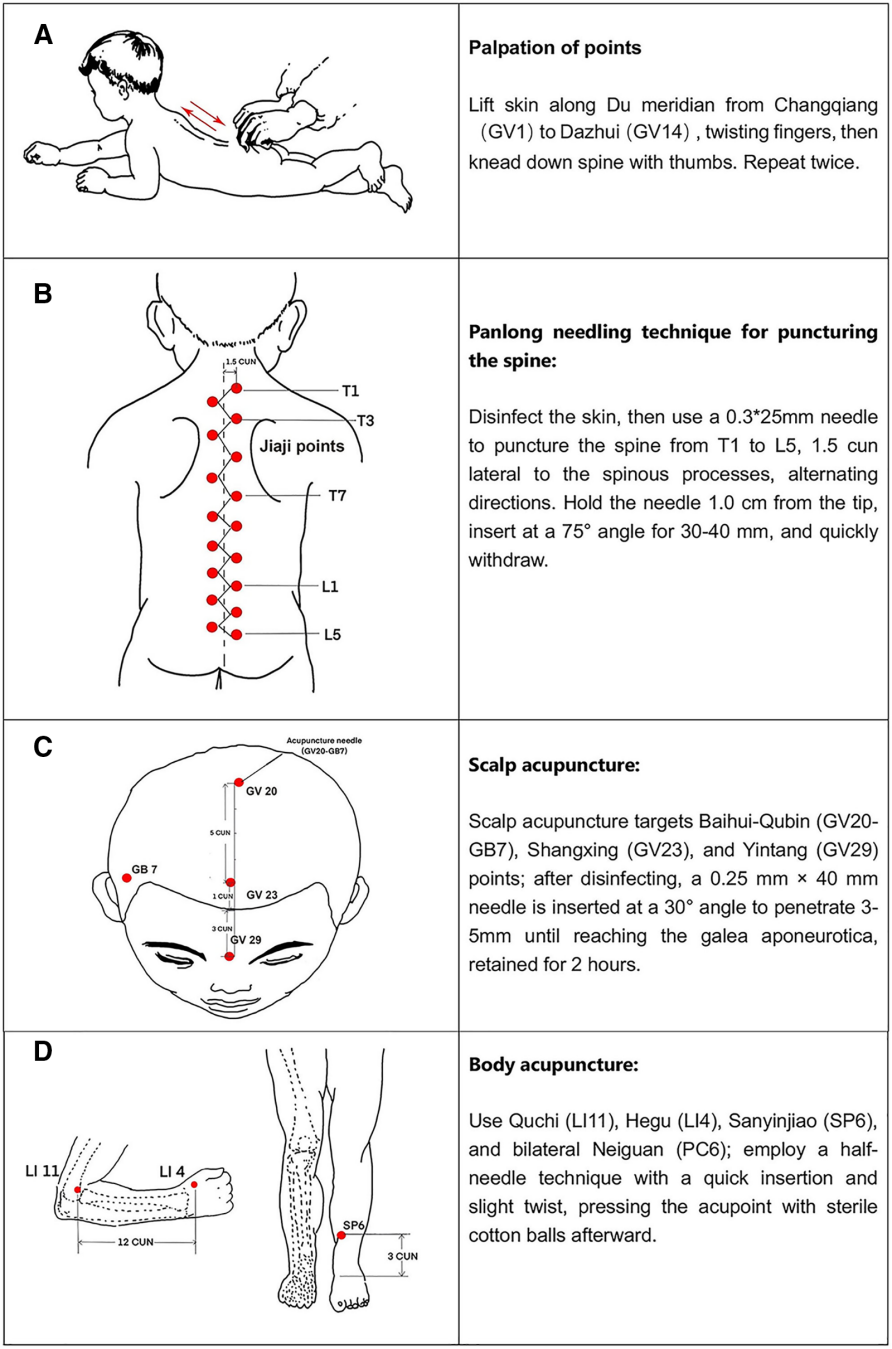
Acupoints and techniques used for treatment: (A) Palpation of points; (B) Panlong needling technique for puncturing the spine; (C) scalp acupuncture; (D) Body acupuncture. “Cun” is a traditional relative length unit in Chinese medicine based on the patient’s body surface landmarks. For example, the distance from the sternal notch to the umbilicus is defined as “8 cun,” with this length divided into equal parts using anatomical landmarks such as joints and wrinkles. T1, T7, T12, L1, and L5 refer to the level segments of the 1st, 7th, and 12th thoracic vertebrae, and the 1st and 5th lumbar vertebrae, respectively. The corresponding Jiaji acupoints are located below the spinous processes of the 1st, 7th, and 12th thoracic vertebrae, and the 1st and 5th lumbar vertebrae, 1.5 cun away from the posterior midline.

Therapeutic method (Fig. 3): Palpation of points (pinching the spine): The patient lies prone; the practitioner warms their hands and relaxes the child’s back muscles. Starting from the Changqiang (GV1) point, use thumb, index, and middle fingers to lift the skin along the Du meridian from bottom to top, with the thumb pushing forward and the index and middle fingers twisting backward, until reaching the Dazhui (GV14) point. Finally, use both thumbs to apply pressure and knead down along the spine. Repeat 2 times. Panlong needling technique for puncturing the spine: After disinfecting the skin, use a 0.3 × 25 mm acupuncture needle (Dong Bang Medical Co., Ltd., Boryung, Korea) to puncture along the spine from T1 to L5 (1.5 cun lateral to the spinous processes). Alternating between up and down and left and right. Hold the needle tip with the thumb and index finger about 1.0 cm from the tip, inserting the needle at a 75° angle to the spine quickly for 30 to 40 mm, then swiftly withdraw the needle, performing the flat tonifying and dispersing technique. Scalp acupuncture: Acupoints: Baihui (GV20)-Qubin (GB7) (bilateral), Shangxing (GV23), Yintang (GV29). After disinfecting the local skin, acupuncture needle (0.25 mm × 40 mm, Dong Bang Medical Co., Ltd., Boryung, Korea) was quick inserted at a 30° angle to the scalp, penetrate 3 mm to 5 mm along the stimulation line until reaching the galea aponeurotica, and retain the needle for 2 hours once qi is obtained. Body acupuncture: Acupoints: Quchi (LI11) on the affected side, Hegu (LI4), Sanyinjiao (SP6); bilateral Neiguan (PC6). Employ a half-needle technique that involves a single-handed twist and quick straight insertion, penetrating the skin approximately 2 to 3 mm, followed by a slight twist and quick withdrawal of the needle. After needle insertion, apply sterile cotton balls to press the acupoint. Acupuncture sessions were conducted once daily for a duration of 5 months.

Ethical approval for this single-patient case report was waived by the Ethics Committee of The First Affiliated Hospital of Dalian Medical University. This exemption is granted as the study involves retrospective analysis of anonymized routine clinical data without additional interventions. Written informed consent was obtained from the legal guardian of the patient for publication of this case report and accompanying images.

## 3. Treatment response

During the treatment, the child patient was conscious, and responsive to sounds and light, and external stimuli sensitively. The muscle tonus of the limbs was normal. The GMFM was used to assess the patients’ gross motor function. After 1 month of treatment, the child still had difficulty bearing weight and grasping with the right upper limb, and crawling remained unstable. After 3 months of treatment, the right upper limbs were able to bear weight briefly, grasp nearby objects with 1 hand, and could crawl short distances. After 5 months of treatment, the child can use the right hand to pick up and return objects while maintaining body balance, and the right limb can support the body during crawling.

The patient’s upper limb function recovery was assessed using the Fine Motor Function Measure (FMFM). After 1 month of treatment, muscle strength in the right upper limb was rated at level IV, with the patient able to move upon prompting and grasp larger toys. After 3 months, muscle strength remained at level IV, but voluntary movement increased; the patient could briefly support their body while lying prone. The patient could also hold a pen and spoon using a 4-finger grasp with the thumb on top, although dexterity was low. By 5 months of treatment, muscle strength in the right upper limb improved to level IV+, and the right thumb was relaxed in an abducted position, allowing the patient to pick up smaller toys, and the thumb and index finger could slowly and accurately touch each other, indicating significant improvement in voluntary movement compared to earlier assessments (Table 1). Telephone follow-ups indicated that the efficacy of the treatment remained stable after 3 months.

**Table 1:** Treatment response.

Treatment duration	Muscle strength	GMFM assessment	FMFM assessment
1 mo	Level IV	Difficulty bearing weight and grasping with the right upper limb, crawling unstable	Able to move upon prompting and grasp larger toys
3 mo	Level IV	Right upper limbs could bear weight briefly, grasp nearby objects with 1 hand, crawl short distances	Voluntary movement increased, could briefly support body while lying prone, hold a pen and spoon with a 4-finger grasp but low dexterity
5 mo	Level IV+	Can use right hand to pick up and return objects while maintaining body balance, right limb can support body during crawling	Muscle strength improved, right thumb relaxed in abducted position, can pick up smaller toys, thumb and index finger could slowly and accurately touch each other, significant improvement in voluntary movement

FMFM = fine motor function measure, GMFM = gross motor function measure.

## 4. Discussion

The diagnosis of pediatric stroke is often delayed, primarily due to postoperative sedation and anesthesia, which can obscure stroke symptoms.^[6]^ Additionally, acute hemiplegia may be less noticeable in newborns. Pediatric strokes frequently present with seizures, and clinical detection can be challenging.^[7]^ This case needs to be distinguished from hypoxic-ischemic encephalopathy (HIE) in newborns, which typically presents with periventricular white matter softening, cerebral artery infarction, paracentral lobule injury, basal ganglia damage, thalamic injury, and selective neuronal necrosis as seen on 2-dimensional ultrasound. Furthermore, the symptoms of central nervous system damage, including cerebral palsy, epilepsy, cognitive impairment, and motor function loss, closely resemble those of pediatric stroke.^[8]^ However, HIE is generally associated with perinatal factors, and diagnostic criteria include persistent neurological symptoms after birth, severe asphyxia, and significant fetal distress.^[9]^ This case does not meet the above criteria and therefore cannot be diagnosed as HIE.

The Panlong acupuncture technique typically refers to the alternating acupuncture of the Huatuo Jiaji points from top to bottom, resembling a dragon coiling on the back, hence the name “Panlong acupuncture technique.” The Huatuo Jiaji points are located 1.5 cun lateral to the midline of the back between the spinous processes of the vertebrae, running parallel from the first lumbar vertebra to the fifth lumbar vertebra, with a total of 34 points, situated between the Du meridian and the bladder channel’s back shu points. The function of the Panlong acupuncture technique is to regulate the qi of the Du meridian and the bladder channel, and it is commonly used in clinical practice to treat spinal diseases, such as ankylosing spondylitis, as well as neuroses and insomnia. But there are few reports on its application in treating brain diseases.

In the case of patients being children, the patient may often struggle to cooperate with needle retention. Therefore, during treatment, a 0.3 × 25 mm acupuncture needle is used with the Panlong needling technique. The needle is quickly inserted and withdrawn at acupoints without retention to achieve a strong sensation. This treatment incorporates the “pinching the spine” technique to locate acupoints, integrating it with the Panlong needling method. The “pinching the spine” technique should be applied gently to the skin and muscles with light pressure. Pinching the spine before needling supports the child’s growth and development. It also unblocks the Du meridian (governing vessel) and enhances the acupuncture sensation.

The “Xingnao Kaiqiao” acupuncture technique, known as “Awakening the brain and opening the spirit” utilizes the acupoints Yintang (GV29), Shangxing (GV23), bilateral Neiguan (PC6), and the affected side’s Sanyinjiao (SP6).^[10]^ The selection of acupoints in the “Xingnao Kaiqiao” acupuncture technique focuses on the concept of regulating the spirit, which encompasses both mental consciousness and the external manifestation of “yang qi.” Here, “spirit” refers to mental consciousness and the external expression of “yang qi,” which energizes the body’s functions. According to TCM, the spinal cord connects to the brain and is crucial for forming the body’s neural network. The “marrow” in the spine corresponds to the Du meridian, and it is also believed that “the kidney governs bone and produces marrow.” When a child has adequate kidney qi, the yang qi can adequately nourish the bones and marrow. Regarding manipulation techniques, all scalp acupoints are superficially needled in this case, as the patient is a child. During insertion, a rotating technique is used 2 to 3 times for both tonification and sedation to stimulate the essence.

The “GV20-GB7” acupuncture technique is frequently used to treat cerebrovascular diseases, such as strokes, and other neurological disorders, also demonstrating promising results for pediatric cerebral palsy.^[11]^ Recent research supports this approach, indicating that prolonged needle retention can regulate glucose metabolism in the brain’s motor areas, activate neural tissues associated with motor functions, facilitate the reconnection of damaged neural networks, and enhance functional recovery.^[12,13]^

After thoroughly assessing the patient’s condition, we chose the Panlong needling technique along with the long retention method for “Xingnao Kaiqiao” scalp acupuncture. The long retention needles at the scalp points aim to improve the brain’s ischemic state and promote the development of the central nervous system. Additionally, we selected the Jiaji points for the Panlong needling technique, which aligns with the TCM concept of the brain connected to the Du meridian.

This study has several limitations, including that it is a single case report without a control group, which means the effectiveness of acupuncture alone in treating this disease cannot be fully established without comparing it to conventional treatment. However, acupuncture is a good adjunctive therapy for patients with poor outcomes from conventional treatment.

## Acknowledgments

The authors thank Macau University of Science and Technology (Faculty of Chinese Medicine) for valuable discussions on clinical acupuncture methodology. We look forward to potential collaborations in future research initiatives.

## Author contributions

**Funding acquisition:** Luling Wang.

**Methodology:** Ruiqiao Guan, Jing Chen.

**Project administration:** Ruiqiao Guan.

**Supervision:** Ruiqiao Guan, Luling Wang.

**Visualization:** Ruiqiao Guan, Jing Chen.

**Writing – original draft:** Ruiqiao Guan.

**Writing – review & editing:** Ruiqiao Guan, Luling Wang, Jing Chen, Shiyu Su, Wei Liu.
